# Compliance with the north American Society of Pacing and Electrophysiology guidelines on amiodarone monitoring in Riyadh, Saudi Arabia: a retrospective charts review study

**DOI:** 10.1186/s40545-020-00235-1

**Published:** 2020-08-18

**Authors:** Bander Ahmed Albassam, Nouf Mohammed Almutairi, Sarah Fahad Aldosari, Hind Saleh Almodaimegh

**Affiliations:** 1 College of Pharmacy, King Saud bin Abdulaziz University for Health Sciences, Riyadh, Saudi Arabia; 2 King Abdullah International Medical Research Center, Riyadh, Saudi Arabia; 3 Pharmacutical Care Services, King Abdualziz Medical City, Riyadh, Saudi Arabia

**Keywords:** Amiodarone, Monitoring, Baseline, Follow-up, Compliance, Adherence, Guidelines

## Abstract

**Background:**

Amiodarone is known for its efficacy as an antiarrhythmic agent; however, its extensive side-effect profile requires careful selection of patients and frequent monitoring. The purpose of this study was to evaluate the performance of the baseline tests before initiating amiodarone therapy and the on-going monitoring based on the North American Society of Pacing and Electrophysiology guidelines recommendations.

**Methods:**

A retrospective descriptive charts review study included all patients who are 18 years of age and older and were started on oral amiodarone with a primary diagnosis of any type of cardiac arrhythmia from January 2016 to December 2018 in King Abdualziz Medical City, Riyadh, Saudi Arabia. The medical charts were reviewed and evaluated based on the performance of the recommended baseline and follow-up of chest X-ray (CXR), liver function test (LFT), thyroid function test (TFT) and electrocardiogram (ECG). The continuous variables were analyzed and presented as mean ± SD and the categorical variables were presented as percentages.

**Results:**

Over the study period, 143 eligible participants on amiodarone therapy were included, with an average of 165 ± 207 days on amiodarone. Of patients, 36.4% had the entire recommended baseline assessments before initiating amiodarone. Our results indicated optimal compliance rates to the baseline tests of CXR (79.7%), LFT (79.7%) and ECG (86.7%). However, there was a lower compliance rate to TFT recommendations (40.6%). The compliance rate to the guideline recommendations related to the follow-up tests was minimal. On-going monitoring performance rates were 47.6% of CXR, 49% of LFT, 54.5% of ECG and 22.4% of TFT.

**Conclusion:**

The compliance with the guideline recommendations related to amiodarone baseline assessments was optimal for all the baseline tests, except for TFT. However, the proportion of patients who received all the recommended baseline assessments was minimal. In addition, the performance of on-going monitoring was suboptimal for all the follow-up tests. Improvements could be made by establishing a local protocol for amiodarone monitoring and pharmacists participating in amiodarone therapy assessments.

## Introduction

Amiodarone is one of the most commonly prescribed antiarrhythmic drugs to treat supraventricular and ventricular arrhythmias [1], as well as controlling atrial fibrillation [2, 3]. Despite its proven efficacy, amiodarone is associated with severe and potentially life-threatening adverse drug reactions that require careful monitoring to ensure safety and optimal benefit with the least risk [4]. The adverse effects of amiodarone include organ toxicities of the liver, thyroid gland, lungs and eyes. The incidence of amiodarone-induced toxicity is relatively high in the general population, including thyroid toxicity in 1–22%, hepatic toxicity in 15–50% and pulmonary toxicity in 2–7% [5]. As a result, several baseline assessments including electrocardiogram (ECG), thyroid function test (TFT), liver function test (LFT), chest X-ray (CXR) and pulmonary function test (PFT) have to be performed before the initiation of amiodarone therapy and frequently monitored to ensure patient safety within the treatment period [4, 6, 7]. These findings emphasize the importance of following the recommended baseline and follow-up tests when initiating amiodarone therapy.

### The rational and significance of the study

Current practice at King Abdualziz Medical City (KAMC) regarding baseline assessments and frequent monitoring of amiodarone related toxicity is unknown. In addition, data on the compliance with laboratory monitoring for patient starting amiodarone therapy in Saudi Arabia are lacking and has not been previously reported. The study aims to assess the compliance with the baseline and follow-up tests recommended by the North American Society of Pacing and Electrophysiology (NASPE) guideline for patients started on amiodarone therapy. The NASPE guideline was published in 2000 and updated in 2007 [4].

## Methods

### Design, setting, participants, and data collection

A retrospective descriptive study that included the patients who received amiodarone therapy with a primary diagnosis of any type of cardiac arrhythmia at KAMC from January 2016 to December 2018. All data were collected from the Best-Care electronic medical records system. The extracted patients data were demographics (age and gender), past medical history, date of amiodarone initiation, the indication of amiodarone therapy, duration of amiodarone therapy and baseline and follow-up laboratory tests performances, including CXR, LFT, TFT and ECG. Initially, PFTs were collected, but due to the negligible number of performances, they were not analyzed and reported in the study. Amiodarone monitoring is defined as performing CXR, ECG, any TFTs including free or total T3, free or total T4, or TSH and any LFTs, which include ALT or AST.

### Exclusion criteria

Excluded participants were patients younger than 18 years of age, patients with a history of abnormal baseline thyroid function (either hypothyroidism or hyperthyroidism), patients with a history of abnormal baseline liver function test defined as a baseline that is three times upper the normal limit (3 × ULN) and patients who were prescribed thyroxin. Abnormal thyroid function is classified as either hypothyroidism or hyperthyroidism based on the following normal ranges which are provided in the Best-Care system: TSH of 0.5–6 uU/ml, Free T4 of 0.7–1.9 ng/dl and Free T3 of 230–619 pg/dl. Abnormal baseline liver function is defined as either ALT that is 3 × ULN of 5–55 U/L or AST that is 3 × ULN of 5–34 U/L.

### Data interpretation

Participants were considered to have a complete functional amiodarone profile in the study if TSH, LFT, CXR and ECG were all performed before the initiation of amiodarone therapy. The appropriate baseline and follow-up monitoring recommended by the NASPE guidelines are provided in Table 1.

**Table 1:** Appropriate baseline and follow-up monitoring recommendations (NASPE) [4]

Monitoring test	Recommendation
CXR	- At baseline - Every 12 months
LFT	- At baseline - Every 6 months
TFT	- At baseline - Every 6 months
ECG	- At baseline - When clinically relevant

### Statistical analysis

The included patients medical records were evaluated for the recommended baseline laboratory monitoring before the initiation of amiodarone and further follow-up monitoring based on the NASPE recommendations. Data are presented as patient and test-specific variables that were entered into an Excel sheet and descriptive statistics were applied to determine the proportion of the patients who received the appropriate amiodarone baseline testing and follow-up monitoring.

## Results

### Sample size and participants characteristics

Overall, the study included 143 eligible participants. The mean age of the participants is 65 ± 14.68 years with males representing 60.1%. Further participants characteristics are listed in Table 2 and Table 3**.**

**Table 2:** Demographics of the study's participants

Demographics	N (%)
**Gender**	
Male	86 (60.1)
Female	57 (39.9)
**Age Group (Years)**	
20 to 35	4 (2.8)
36 to 50	20 (13.9)
51 to 65	46 (32.2)
66 to 80	52 (36.4)
More than 80	21 (14.7)

**Table 3:** Past medical history of the study's participants

Comorbidity N (%)	
**Hypertension**	**Lung disease**
108 (75.5)	32 (22.4)
**Diabetes mellitus**	**Renal disease**
94 (65.7)	49 (34.3)
**Dyslipidemia**	**Liver disorder**
48 (33.6)	11 (7.7)
**Myocardial infraction**	**Malignancy**
47 (32.9)	18 (12.6)
**Coronary artery disease**	**Gout**
89 (62.2)	2 (1.4)

### Amiodarone indications and length of treatment

Amiodarone was mainly indicated for atrial fibrillation in 108 (75.52%) of participants. Into lesser extents, it was initiated for ventricular tachycardia, atrial flutter, premature ventricular contraction, atrial tachycardia and ventricular fibrillation in 16 (11.20%), 12 (8.40%), 4 (2.80%), 2 (1.40%) and 1 (0.70%) patient(s), respectively. The patients were on amiodarone therapy for an average of 165 ± 207 days.

### The compliance with the baseline tests recommendations

There were optimal compliance rates to the NASPE recommendations in terms of preforming the baseline monitoring of CXR, LFT and ECG in 114 (79.7%), 114 (79.7%) and 124 (86.7%) patients, respectively. However, baseline assessments of thyroid function were surprisingly suboptimal, as they were only performed to 58 (40.6%) patients. Overall, only 52 (36.4%) patients received the complete baseline monitoring tests.

### The compliance with the follow-up tests recommendations

The compliance with the guidelines recommendations was low in terms of performing the on-going monitoring examinations. Low performance rates were observed for CXR, LFT and ECG follow-ups in 68 (47.6%), 70 (49%) and 78 (54.5%) patients, respectively. An extremely lower performance rate was for TFT, as it was received by 32 (22.4%) patients only.

## Discussion

This study aimed to assess the compliance rate to the NASPE guidelines recommendations in terms of baseline assessments and follow-up related to amiodarone therapy at KAMC, Riyadh, Saudi Arabia. Locally, similar studies are lacking; raising the necessity for the current study. The compliance was assessed by evaluating the rate of CXR, LFT, TFT and ECG performances at baseline, prior to amiodarone therapy initiation, and as ongoing follow-up. The compliance rate to the baseline of CXR and LFT recommendations in our institution was 79.7%. Among the patients on chronic amiodarone therapy, 86.7% had ECG performed and 40.6% had TFT measured. Distinguishably, TFT baseline is rarely evaluated, which is of concern. Despite thyroid toxicity being one of the most amiodarone reported adverse effects, it appears that there is a lack of awareness in our institution on TFT monitoring importance [5]. Comparing our results to other studies, Lavon et al., (2019) found that 97% of patients were evaluated for thyroid and liver functions prior to amiodarone treatment. Forty-eight percent of the patients performed CXR and all patients (100%) conducted ECG [8]. According to a study performed by Rankin et al., (2017), the recorded adherence was 46% for liver and 28% for thyroid baseline assessments [9]. The current study found a low rate of CXR follow-up (47.6%), which is consistent with a previous publication (50%) [6]. A similar result was found regarding LFT follow-up performance (49%), which is in alliance with multiple previous publications (40.5, 52 and 46%) [6, 7, 9]. TFT follow-up was conducted for only 22.4% of the patients included in our study, which is of concern, but consistent with several published studies reporting 32, 20, 16.5 and 28% of TFT follow-up performances [6, 7, 9, 10]. The performance rate of on-going ECG examination was low (54.5%), but in line with the current literature (55%) [8]. Overall, the compliance rate to the amiodarone monitoring recommendations was partially low at KAMC. The suboptimal compliance rates to the follow-up recommendations are potentially due to the lack of a written guideline used as a reference standard for amiodarone monitoring practice at KAMC. The local practice of amiodarone monitoring rather based on guidelines that are not physically posted or uploaded in the electronic system, physician expertise and clinical pharmacists interventions. It is noteworthy that the clinical pharmacists at KAMC have a limited role in amiodarone follow-up. Usually, patients undergo follow-up in the outpatient clinics, where clinical pharmacists have limited role in, which might also contribute to the insufficient compliance to the follow-up recommendations. The concern of suboptimal amiodarone monitoring is global. Therefore, multiple studies evaluated the impact of enormously involving pharmacists to overcome the issue. In a cohort study conducted by Samuel G. Johnson et al., (2010), the performance rates of baseline, 6 and 12 months follow-up of ALT were significantly higher (44% vs. 69, 76% vs. 86, and 69% vs. 84%) in the patient group monitored collaboratively by clinical pharmacists, respectively. The performance rates of baseline and 1 year follow-up of ECG were also significantly higher (58% vs. 76 and 75% vs. 89%) in the group monitored collaboratively by pharmacists, respectively. Also, there were significantly fewer amiodarone related adverse events (21 vs. 48) in the group monitored by pharmacists compared to the control group [11]. Another prospective randomized blinded study achieved 56% of improvements on the adherence to amiodarone monitoring recommendations in the group monitored by pharmacists along with a collaborative team [12].

### Study’s limitations

The limitations of the study include the retrospective nature of the study, performing the study in a single center, the limited sample size and the possibility of overestimating guideline compliance rates as the purpose of some of the performed tests is not certainly intended for amiodarone monitoring.

## Conclusion

The compliance with the NASPE guidelines on amiodarone baseline monitoring of CXR, LFT, and ECG was optimal. However, the recommendation on baseline TFT assessment was minimally adhered. The number of patients who received all the recommended baseline tests was minimal. The study indicated a suboptimal amiodarone follow-up in the local setting. Opportunities to enhance amiodarone monitoring exist. Improvements could be established by implementing a local amiodarone monitoring protocol based on international guidelines. In addition, based on the literature, involving pharmacists in amiodarone monitoring is proven to optimize amiodarone monitoring practice.

## Abbreviations

NASPE: North American Society of Pacing and Electrophysiology
KAMC: King Abdualziz Medical city
CXR: Chest X-ray
LFT: Liver function test
TFT: Thyroid function test
ECG: Electrocardiogram
PFT: Pulmonary function test
T3: Triiodothyronine
T4: Thyroxine
TSH: Thyroid stimulating hormone
ALT: Alanine aminotransferase
AST: Aspartate aminotransferase
3 × ULN: Three times the upper limit of normal
